# GATA3 Exerts Distinct Transcriptional Functions to Regulate Radiation Resistance in A549 and H1299 Cells

**DOI:** 10.1155/2022/9174111

**Published:** 2022-08-10

**Authors:** Rui Wang, Junxuan Yi, Hui Gao, Xinfeng Wei, Lihong Shao, Mingwei Wang, Weiqiang Xu, Xiaoshu Yin, Yannan Shen, Zhicheng Wang, Wei Wei, Shunzi Jin

**Affiliations:** ^1^NHC Key Laboratory of Radiobiology, School of Public Health, Jilin University, Changchun, Jilin, China; ^2^Department of Orthopedics, The First Hospital of Jilin University, Changchun, Jilin, China; ^3^Department of Radiation Oncology, The First Bethune Hospital of Jilin University, China; ^4^Affiliated Dongguan People's Hospital, Southern Medical University, Dongguan, Guangdong, China

## Abstract

**Background:**

Radiation resistance of lung cancer cells is a vital factor affecting the curative effect of lung cancer. Transcription factor GATA3 is involved in cell proliferation, invasion, and migration and is significantly expressed in a variety of malignancies. However, the molecular mechanism governing GATA3 regulation in lung cancer cells' radiation resistance is unknown.

**Methods:**

Radiation-resistant cell models (A549-RR and H1299-RR) were made using fractionated high-dose irradiation. Use clone formation, CCK-8, F-actin staining, cell cycle detection, and other experiments to verify whether the model is successfully constructed. Cells were transiently transfected with knockdown or overexpression plasmid. To explore the relationship between GATA3/H3K4me3 and target genes, we used ChIP-qPCR, ChIP-seq, and dual luciferase reporter gene experiments. Xenograft tumor models were used to evaluate the effect of GATA3 depletion on the tumorigenic behavior of lung cancer cells.

**Results:**

We report that transcription factors GATA3 and H3K4me3 coactivate NRP1 gene transcription when A549 cells develop radiation resistance. However, the mechanism of radiation resistance in H1299 cells is that GATA3 acts as a transcription inhibitor. The decrease of GATA3 will promote the increase of NRP1 transcription, in which H3K4me3 does not play a leading role.

**Conclusions:**

GATA3, an upstream transcriptional regulator of NRP1 gene, regulates the radioresistance of A549 and H1299 cells by opposite mechanisms, which provides a new target for radiotherapy of lung cancer.

## 1. Introduction

Radiotherapy is one of the most effective cancer therapy approaches that involves complex biological processes to induce cancer cell death [1–3]. The factors that cause cancer patients to produce radiotherapy resistance during treatment are diverse. Molecular mechanisms of radioresistance remain unclear.

GATA binding protein-3 (GATA3) is a transcription factor consisting of 443 or 444 amino acids with two activation domains and two zinc finger structures [4, 5]. GATA3 affects target gene expression and triggers the proliferation and differentiation of specific immune cells [6, 7]. GATA3 is expressed in various tissues and cell types, especially breast, central nervous system, kidney, hair follicle skin, helper T cells (Th2), lymphocytes, and so on [8]. High GATA3 expression in lung adenocarcinoma has also been considered an evaluation factor of poor prognosis [4, 9–11]. However, its function remains elusive.

Neuropilin-1 (NRP1) is a transmembrane protein overexpressed in advanced human tumors, typically exhibiting growth-promoting functions in cancer cells [12, 13]. As a coreceptor for vascular endothelial growth factors (VEGFs), NRP1 has been shown to interact with the cell surface epidermal growth factor receptor (EGFR) to promote intracellular signaling. The mechanisms modulating *NRP1* expression in cancer cells are controversial. For example, growth factors and stimulation of the RAS-MAPK signaling pathway may stimulate NRP1 transcription [14, 15]. On the other hand, *NRP1* transcripts are proposed targets for miRNA-338 [16] and other miRNAs. At present, research on NRP1 is involved in many fields such as angiogenesis, the hematopoietic system, immune system, and tumor occurrence and development [17, 18]. Previous studies have supported the notion that elevated *NRP1* expression in tumors correlates with poor outcomes. On the other hand, NRP1 is associated with radiation resistance [19]. However, the molecular mechanism through which NRP1 plays a crucial role in the formation of radiation resistance in lung cancer cells is unknown; in particular, more research into transcription factor regulatory mechanisms is needed.

KMT2B (Lysine Methyltransferase 2B), also known as MLL2 (Mixed Lineage Leukemia 2), belongs to the family of mammalian histone H3 lysine 4 (H3K4) methyltransferases and forms a protein complex with WRAD (WDR5, RbBP5, ASH2L, and DPY30), Host Cell Factors 1/2 (HCF 1/2), and Menin. The MLL2 complex is responsible for H3K4 trimethylation (H3K4me3) on specific gene promoters and nearby cis-regulatory sites, regulating bivalent developmental genes as well as stem cell and germinal cell differentiation gene sets [20].

The research results of this project suggest that the present ChIP-seq and RNA-seq analyses reveal that *NRP1* is a key gene directly regulated by GATA3 and H3K4me3 that is involved in the formation of tumor cell radiation resistance. Moreover, radiation resistance mechanisms of the two lung cancer cell types are different. In A549-RR cells, GATA3 and H3K4me3 coregulate *NRP1* expression positively, while GATA3 represses NRP1 expression in H1299-RR cells.

## 2. Methods and Materials

### 2.1. Cell Culture and Transfection

Human lung cancer cell lines A549 and H1299 were purchased from Cell Bank Type Culture Collection of the Chinese Academy of Sciences (Shanghai, China). A549 cells were maintained in DMEM/high glucose (Gibco, USA). H1299 cells were maintained in RPMI-1640 medium (Gibco, USA). 10% Fetal Bovine Serum (HyClone, USA), 100 U/ml penicillin, and 100 *μ*g/ml streptomycin were added to the medium. Cell growth environment was in a humidified CO_2_ incubator at 37°C and 5% CO_2_. Cell lines were regularly confirmed to be free from mycoplasma contamination using MycoAlert Detection Kit (Lonza). GATA3 small interfering RNA (siRNA, GenePharma, China) with the corresponding control RNA (siNC), recombinant plasmid overexpressing GATA3 with the empty pcDNA3.1(+) vector (PLL, Nanjing, China), the lentiviral shRNA against human GATA3 and NRP1 (PLL, China) with corresponding control RNA (shNC), or recombinant plasmid overexpressing NRP1 with the empty PLNCX2 vector (Sigma, USA) were transfected into cells. The transfection was carried out according to the manufacturer's instructions using the Lipofectamine 2000 transfection reagent (Invitrogen, USA). Table S1 shows the sequences of the shRNA and siRNA oligonucleotides.

### 2.2. Dose-Gradient Irradiation

A549 and H1299 cells were performed at a rate of 1.02 Gy/min for A549 cells and 0.75 Gy/min for H1299 cells [21] at room temperature using an X-ray generator (Model X-RAD320iX, USA). They were exposed to 6 Gy every time, 5 times, 30 Gy in total. Cells were passaged 4 times or more for subsequent experiments. For the mouse radiosensitivity experiment, a radiation dose of 20 Gy was administered when the xenografts reached an average volume of 200 mm^3^ [22]. Finally, mice were euthanized at prespecified times.

### 2.3. Mouse Irradiation Study

Six-week-old BALB/c nude mice were purchased from Beijing Huafukang Biotechnology Company (China). Using lentiviral plasmids (PLL Company, Nanjing, China). We constructed stable knockdown GATA3 cell models in A549 and H1299 cells. Table S1 shows the sequences of the shNC and shGATA3. A total of 1 × 10^6^ cells were resuspended in 100 *μ*l of PBS and injected subcutaneously into the right hind legs of mice. All animals were kept in laboratory animal centers, in accordance with the regulations of the Medical Ethics Committee. Every two days, body weight and tumor volume measures were taken. All animals were sacrificed 14 days after irradiation and their excised tumors were weighed. Tumor volume (mm^3^) = *a*^2^ × *b*/2 (*a*: length (mm), *b*: width (mm)). The BALB/c nude mice used in this experiment had passed the quality test, and the license number of Beijing Huafukang Company is SCXK (Jing) 2019-0008.

### 2.4. Colony Formation Assay

1000 cells were seeded into 6-well plates. The next day, cells were irradiated with X-rays of 0, 2, 4, 6, 8 Gy. After 2 weeks, cell colonies were washed 3 times with PBS for 3 min each, fixed with 4% paraformaldehyde for 30 min, and stained with crystal violet (Solarbio, Beijing, China) for 30 min.

### 2.5. Cell Cycle Analysis

The cells were made into one cell suspension, cleaned twice with cold PBS, added with 300 *μ*l of PBS cell suspension, added with 150 *μ*l of 75% ethanol, fixed at 4°C for 2 hours, washed twice with PBS, added with an appropriate amount of PI, placed in the dark for 15 min, and subjected to flow cytometry analysis. The machine type of the flow analyzer is a FACSCalibur flow cytometer, and the company is BD Biosciences (USA).

### 2.6. Quantitative Real-Time PCR

RNA was remoted with TRIzol (Invitrogen, USA) and reverse transcribed to produce cDNA (TaKaRa Reverse Transcription Kit, Dalian, China) according to the manufacturer's protocol. With GAPDH as the internal control, qRT PCR was performed by the TB-Green assay (Fluorescence Quantitation Kit, TaKaRa). Relative quantification of gene expression was computed using the method of 2^-*ΔΔ*CT^. The sequences of the qRT-PCR primers are listed in Table 1.

### 2.7. Immunofluorescence

The cells were cleaned with PBS three times. Then, we conducted a more in-depth exploration. After washing, the cells were subjected to 30 min of membrane permeation with 0.5% Triton X-100, washed with PBS, and then sealed with 10% BSA for 1 h. Take out the blocking solution, put it directly into the wet box, drip the diluted primary antibody, and incubate overnight at 4°C. The next day, after rewarming the wet box at room temperature for 30 min, drip the diluted fluorescent secondary antibody, incubate at room temperature in the dark for 1 h, wash with PBS, drop the sealing agent containing DAPI, store it in a dry cassette at 4°C, and take photos with a fluorescence microscope.

### 2.8. Western Blotting

The total protein sample was prepared using the NP-40 (Beyotime, Shanghai, China). For western blot analysis, identical amounts of protein were resolved by SDS-PAGE, moved to PVDF membranes, immunoblotted with primary (NRP1 1 : 1000; Flag 1 : 1000; GATA3 1 : 1000) and secondary antibodies (Rabbit antibody 1 : 10000; Mouse antibody 1 : 50000), and detected using chemiluminescence (Pierce ECL kit, Thermo Fisher Scientific). The information about primary and secondary antibodies used in this article is listed in Table 2.

### 2.9. Immunohistochemistry (IHC)

The experiment was carried out according to the immunohistochemical kit (purchased from Maixin, Fujian, China). After staining the tissue sections, mount them and take pictures after drying.

### 2.10. Chromatin Immunoprecipitation-Quantitative PCR (ChIP-qPCR)

The sequences of NRP1 gene promoter region were queried by bioinformatics and matched with the GATA3 protein. There were 11 sequences with a matching score of more than 90%, and the optimal binding sequence was selected to design primers. The experimental steps of ChIP-qPCR were carried out in strict accordance with the kit instructions (purchased from ACTIVE-MOTIF, USA). The unique primer sequences are listed in Table 3.

### 2.11. Chromatin Immunoprecipitation Sequencing (ChIP-seq)

For ChIP-Seq, the Shenzhen ACE Gene Company was responsible for sample preparation and sequencing analysis. Raw reads were aligned to the human refence genome (assembly hg19) using Bowtie 2 [23] with default parameters. Peaks were called with MACS2 [24] with default parameters. The colocalization of GATA3 and H3K4me3 peaks was performed using the bed tool [25] intersect utility. Gene set enrichment analysis of overlapping regions for GATA3 and H3K4me3 was performed with the web app GREAT [26] with default settings.

### 2.12. Plasmid Transfection and Luciferase Reporter Assays

For luciferase assays to test the interaction between GATA3 and the NRP1 promoter, A549 cells were transiently cotransfected with 100 ng of pGL3-Basic or NRP1 promoter plasmid and 50 ng of pRL-TK, or its negative control using Lipofectamine 3000 (Invitrogen, USA) in 24-well plates. Then, 48 h after transfection, the luciferase activities were tested using a Dual-Luciferase Reporter Assay according to the E1910 Kit (Promega, USA).

### 2.13. Statistical Analysis

The experimental data in this experiment were statistically analyzed by SPSS 24.0 software, and the obtained data were expressed by means ± standard deviation. The statistical analyses of the experiment data were performed by using a two-tailed Student's paired *t*-test and one-way ANOVA. Statistical significance was assessed at least three independent experiments. *P* < 0.05 considered the difference to be statistically significant.

## 3. Results

### 3.1. Establishment of Radiation-Resistant Cell Model

**Figure 1:**
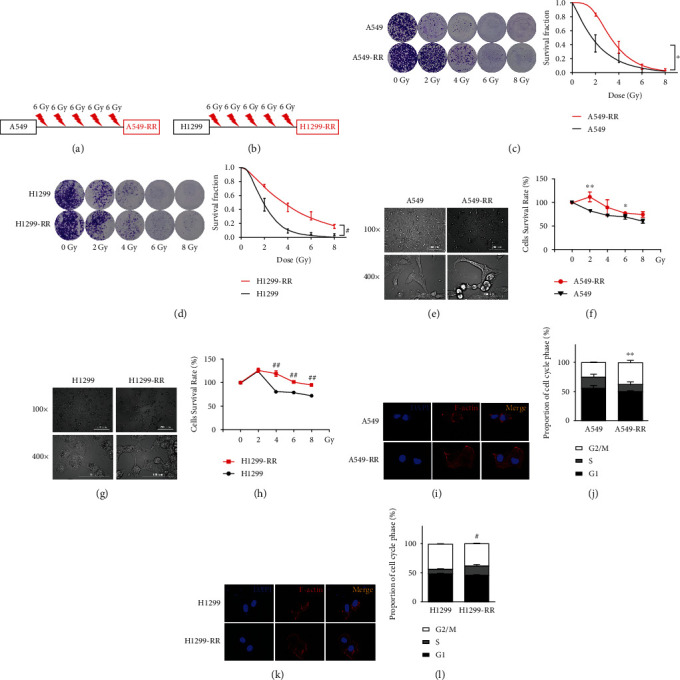
A549-RR and H1299-RR cells are resistant to radiation. (a, b) The strategy of A549 and H1299 radiation resistance cells. (c, d) Cell Counting Kit-8 assay was performed to detect the viability and proliferation of the cells. The survival rate was normalized to 0 Gy. (e, g) Cell morphology was observed by light microscope. (f, h) Colony formation assay was performed to detect the viability of the cells. The survival rate was normalized to the 0 Gy group. (i, k) Double staining of cells for F-actin (red) and for cell nuclei with DAPI (blue). (j, l) Cell cycle distribution of each group. Results were presented as mean ± SD of three independent experiments (*n* = 3; ^∗^*P* < 0.05, ^∗∗^*P* < 0.01 vs. A549; ^#^*P* < 0.05, ^##^*P* < 0.01 vs. H1299; two-tailed *t*-test).

The radioresistant cell lines A549-RR and H1299-RR were created by irradiating the parental A549 and H1299 cell lines with a dose gradient at a high dose of 30 Gy (Figures 1(a) and 1(b)). As the radiation dose increased, we discovered that the survival fraction in A549-RR and H1299-RR cells was substantially higher than in A549 and H1299 cells (Figures 1(c) and 1(d)). Furthermore, A549-RR and H1299-RR cells were larger and had longer pseudopodia compared to parental cells (Figures 1(e) and 1(g)). Next, compared to the parental cells, A549-RR and H1299-RR cells demonstrated a significantly increased survival rate (Figures 1(f) and 1(h)). According to the point-and-click multitarget model, the higher the Dq value, the stronger the ability to repair the sublethal damage of cells. Therefore, the clone formation experiment results confirmed that A549-RR and H1299-RR have stronger damage repair ability (Table S2). The development of radiation resistance resulted in a diffuse cytoplasmic distribution of F-actin (Figures 1(i) and 1(k)). Radiation can cause a different cell cycle arrest [27]. We found that A549-RR cells had G2/M phase arrest and H1299-RR cells had S phase arrest compared with their parents (Figures 1(j) and 1(l)). These results indicate that both A549-RR and H1299-RR cells are resistant to radiation.

### 3.2. GATA3 and NRP1 Are Involved in the Formation of Radioresistance in Lung Cancer Cells

**Figure 2:**
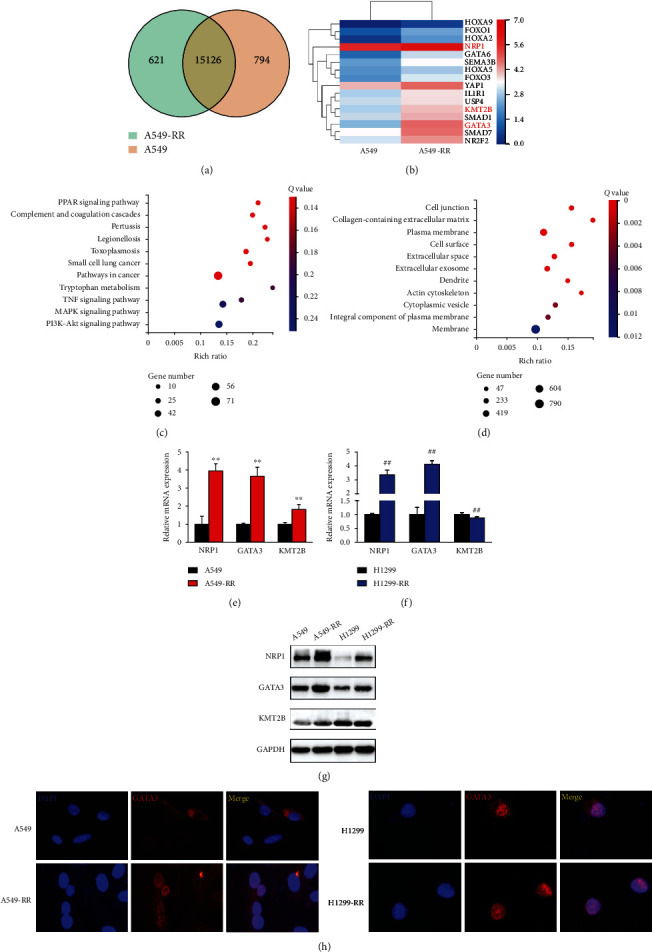
GATA3 and NRP1 are overexpressed in A549-RR and H1299-RR cells. (a) Venn diagram shows the overlap of genes between A549-RR (green) and A549 (yellow). (b) Hierarchical clustering of genes altered by A549 and A549-RR cells. (c) Pathway analysis of differential genes arranged into functional groups. (d) Genes differentially expressed were selected for gene ontology analysis. (e, f) Verification of RNA-seq results through qRT-PCR analysis of the indicated genes in A549, A549-RR, H1299, and H1299-RR cells (*n* = 3; ^∗^*P* < 0.05, ^∗∗^*P* < 0.01 vs. A549; ^#^*P* < 0.05, ^##^*P* < 0.01 vs. H1299; two-tailed *t*-test). (g) Expressions of NRP1, GATA3, and KMT2B were measured using western blotting (*n* = 3). (h) Immunofluorescence staining of cells for GATA3 (red) or NRP1 (red) and for cell nuclei with DAPI (blue) (*n* = 3).

To study the mechanism of radiation resistance, we performed RNA-sequencing. According to the results of RNA-sequence, there were 15,126 genes coexpressed by A549 and A549-RR cells (Figure 2(a)). Heatmap analysis showed that *NRP1*, *GATA3*, and *KMT2B* were the upregulated genes (Figure 2(b)). KMT2B is a member of the H3K4 histone methyltransferase (HMT) family, which can catalyze the trimethylation of histones. Kyoto Encyclopedia of Genes and Genomes pathways were studied for each module which was performed to investigate the signaling mechanisms. The cancer pathways, PI3K-AKT pathway [28], and MAPK pathway were found to be overrepresented in the dysregulated genes (Figure 2(c)). Indeed, differential genes were enriched in the GO biological processes related with the plasma membrane (Figure 2(d)), according to Gene Ontology (GO) analysis. Next, we checked the RNA-seq results and discovered that gene expression in A549 and A549-RR cells matched the sequencing results (Figure 2(e)). In H1299 and H1299-RR cells, however, the mRNA expression of NRP1 and GATA3 rose whereas the mRNA expression of KMT2B decreased (Figure 2(f)). Immunofluorescence results showed that GATA3 nucleation was observed in both radiation-resistant models (Figure 2(h)).

### 3.3. GATA3 Positively Regulates NRP1 Expression in A549-RR Cells but Represses NRP1 Expression in H1299-RR Cells

**Figure 3:**
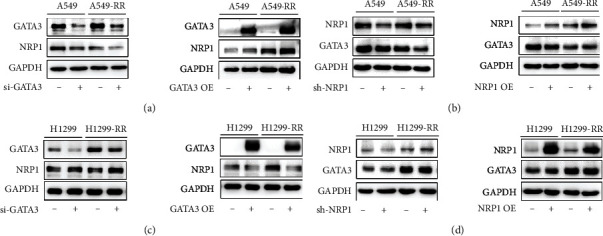
GATA3 positively regulates NRP1 expression in A549-RR cells but represses NRP1 expression in H1299-RR cells. (a) The protein expression of GATA3 and NRP1 involved in A549 and A549-RR cells transfected with negative control siNC, siGATA3, vector, or pcDNA3.1(+)-GATA3. (b) The protein expression of GATA3 and NRP1 involved in A549 and A549-RR cells transfected with negative control shNC or shNRP1 and vector or pLNCX2-NRP1. (c, d) The GATA3 and NRP1 protein level were downregulated or upregulated in H1299 and H1299-RR cells. Cells were transfected for 48 hours and harvested for analysis.

To begin to understand the role of GATA3-mediated transcriptional regulation, we first designed siRNAs to specifically knock down the expression of GATA3 (Figure S1). GATA3 and NRP1 were knocked down and overexpressed using transient transfection of siGATA3, pcDNA3.1(+)-Flag-GATA3, pSIREN-RetroQ-shNRP1, and pLNCX2-NRP1 plasmids. The qRT-PCR results showed that in A549 and A549-RR cells, the mRNA expression of NRP1 decreased after GATA3 knockdown and increased after GATA3 overexpression (Figure S2). Knockdown of GATA3 downregulated the expression of the NRP1 protein; overexpression of GATA3 upregulated the NRP1 protein (Figure 3(a)). On the other hand, knockdown or overexpression of NRP1 showed no obvious change in the GATA3 protein (Figure 3(b)), indicating that NRP1 does not directly affect the expression of GATA3. Thus, GATA3 can positively regulate NRP1 in A549 and A549-RR cells.

Conversely, the phenomenon in H1299 and H1299-RR cells was the opposite compared to that in A549 cells. The mRNA expression of NRP1 increased after GATA3 knockdown and decreased after GATA3 overexpression in H1299 cells and H1299-RR cells (Figure 3(c)). However, there was no significant change in the expression of GATA3 after knockdown or overexpression of NRP1 in other cells (Figure 3(d)). Taken together, these results indicated that GATA3 negatively regulates NRP1 in H1299 and H1299-RR cells. At the same time, we also detected the mRNA expression levels of GATA3 or NRP1 after knockdown or overexpression and found no obvious trend (Figure S3). Therefore, the regulation of GATA3 on NRP1 mainly reflects its protein expression.

### 3.4. GATA3 Is Located Upstream of the *NRP1* Gene to Regulate Transcription

**Figure 4:**
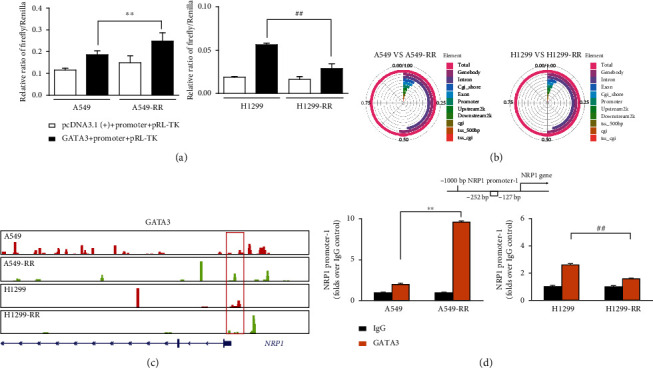
GATA3 regulates the expression of NRP1 target gene. (a) Luciferase values in A549, A549-RR, H1299, and H1299-RR cells cotransfected with pcDNA3.1(+), pcDNA3.1(+)-GATA3, or *NRP1* promoter sequence and pRL-TK measured by a dual-luciferase reporter assay. (b) Genomic distribution of GATA3 targets, based on ChIP-seq analysis. (c) ChIP-seq gene tracks show the binding locations of transcription factor GATA3 at the *NRP1* gene locus in A549, A549-RR, H1299, and H1299-RR cells. Red box indicates the region associated with GATA3 signals in four kinds of cells. (d) ChIP-qPCR with IgG and GATA3 antibody in A549, A549-RR, H1299, and H1299-RR cells, followed by qRT-PCR with primers of the *NRP1* promoter-1 region (-252~-127) specific to the GATA3. Data were shown as means ± SD from three independent experiments (*n* = 3; ^∗^*P* < 0.05, ^∗∗^*P* < 0.01 vs. A549; ^#^*P* < 0.05, ^##^*P* < 0.01 vs. H1299; two-tailed *t*-test).

In order to explore whether GATA3 regulates the transcription of the *NRP1* gene, we constructed a recombinant plasmid of the promoter region of the *NRP1* gene with the pGL3 basic vector. Dual-luciferase reporter assays confirmed that GATA3 promotes activation of the *NRP1* promoter in A549-RR cells, while the ability of GATA3 to activate the *NRP1* promoter was weakened in H1299-RR cells (Figure 4(a)). We analyzed the whole genome distribution of GATA3 target genes in two radiation resistant cells and found that the target genes were widely distributed, including exons, promoters, and so on (Figure 4(b)). In the ChIP-seq analysis, a binding event between GATA3 proteins was observed upstream of the *NRP1* locus (Figure 4(c)). Next, ChIP-qPCR analysis was performed using A549, A549-RR, H1299, and H1299-RR cells and specific antibodies against GATA3 and IgG for selected *NRP1* genes. The results showed a strong enrichment of GATA3 on the gene promoters, validating the ChIP-seq results (Figure 4(d)). These results indicated that *NRP1* is a high-confidence target gene downstream of GATA3 in lung cancer cells.

### 3.5. H3K4me3 and GATA3 Jointly Regulate the Transcription of NRP1 Gene

**Figure 5:**
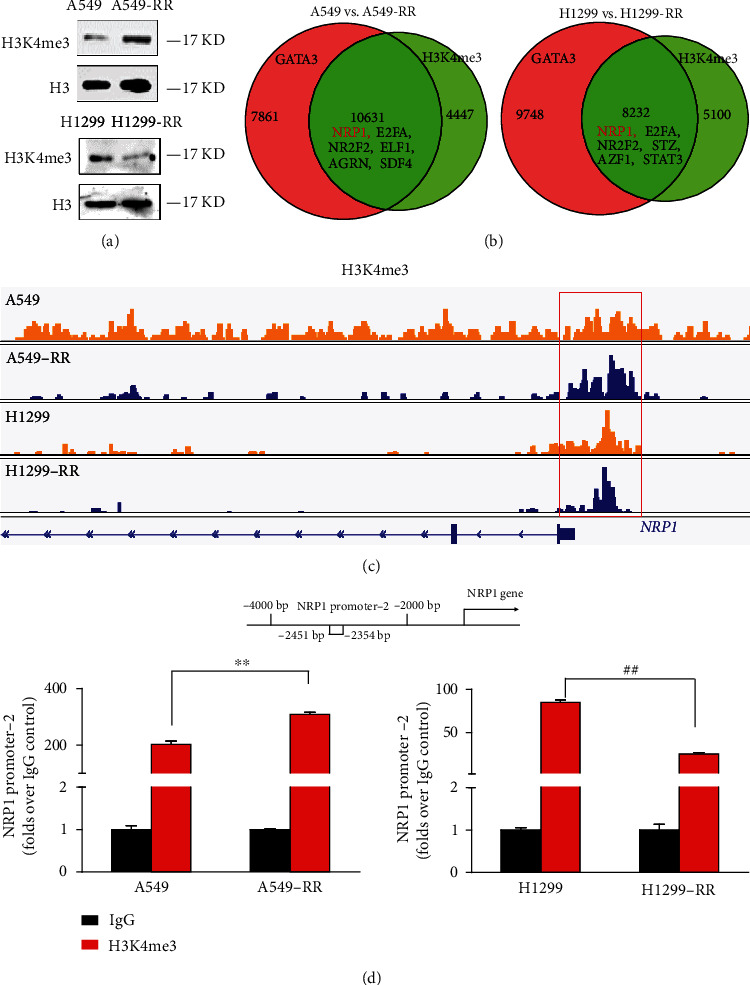
H3K4me3 obviously bind to the NRP1 promoter region. (a) The levels of H3K4me3 modifications in histone extracts were determined via western blotting. (b) Venn diagram showing overlapping genes bound by GATA3 and H3K4me3 in A549, A549-RR, H1299, and H1299-RR cells. (c) ChIP-seq gene tracks show the binding locations of H3K4me3at the *NRP1* gene locus in A549, A549-RR, H1299, and H1299-RR cells. Red box indicates the region associated with H3K4me3 signals in four kinds of cells. (d) ChIP-qPCR with IgG and H3K4me3 antibody in A549, A549-RR, H1299, and H1299-RR cells, followed by qRT-PCR with primers of the *NRP1* promoter-2 region (-2354~-2451) specific to the H3K4me3. Data are shown as means ± SD from three separated experiments (*n* = 3; ^∗^*P* < 0.05, ^∗∗^*P* < 0.01 vs. A549; ^#^*P* < 0.05, ^##^*P* < 0.01 vs. H1299; two-tailed *t*-test).

In RNA-seq results, we found that H3K4me3 methyltransferase KTM2D was upregulated in A549-RR cells, so we speculated that H3K4me3 was likely to participate in the formation of radiation resistance. In support of this finding, we examined the expression of H3K4me3 in two radiation resistance models; western blot analysis showed that A549-RR cell lines had a high expression level of H3K4me3. However, H1299-RR cell lines displayed a low H3K4me3 expression level (Figure 5(a)).

Then, how does H3K4me3 affect radiation resistance? In ChIP-sequence analysis, we found that there were 10631 and 8232 target genes jointly regulated by GATA3 and H3K4me3, respectively, in the two radiation resistance models. Surprisingly, NRP1 was the common target gene, E2FA, NR2F2, and so on were included (Figure 5(b)). We next analyzed the chromatin status at the NRP1 target locus in lung cancer cells and found that *NRP1*-enriched regions were frequently associated with an active histone mark (H3K4me3). Furthermore, the enrichment of H3K4me3 in the promoter region of the NRP1 gene in the two radiation resistant models was different. In A549-RR cells, this enrichment increased, whereas in H1299-RR cells, it reduced (Figure 5(c)). Finally, we verified the enrichment of H3K4me3 according to the ChIP-seq site (-2354~-2451). The results showed that H3K4me3 was indeed enriched here, which also confirmed the results of ChIP-seq (Figure 5(d)).

### 3.6. GATA3 Knockdown Can Enhance A549-RR Cell Radiosensitivity

**Figure 6:**
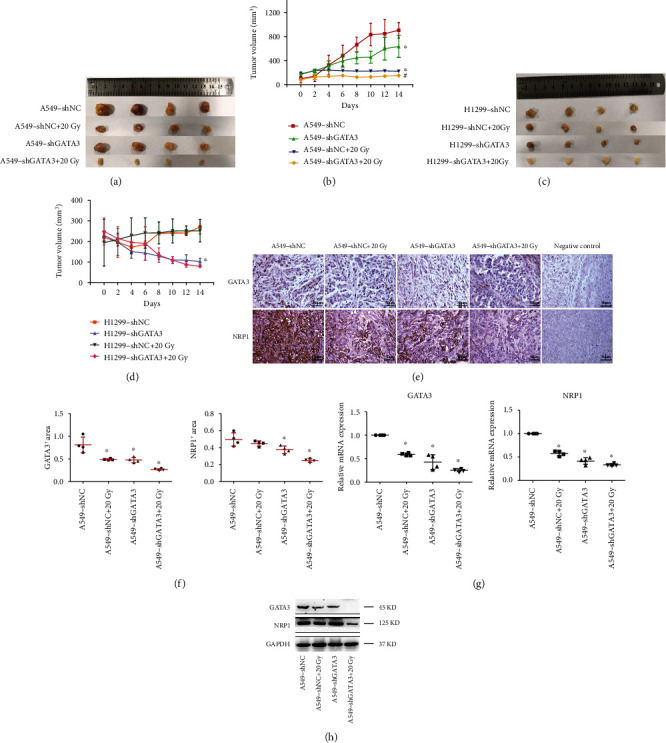
Knockdown of GATA3 can enhance the radiosensitivity of A549-RR cells. (a, c) Cells were injected subcutaneously into nude mice. Mice were exposed to 20 Gy of radiation when the tumor volume reached approximately 200 mm^3^.The tumor was removed 14 days after irradiation. (b, d) The average volumes of the tumors were measured every 2 days (*n* = 4, ^∗^*P* < 0.05 vs. A549-shNC or H1299-shNC, ^#^*P* < 0.05 vs. A549-shNC+20 Gy). (e) IHC staining for GATA3 and NRP1 proteins in tumors of the four groups at 14 days. Scale bars, 50 *μ*m. (f) Quantification of GATA3 and NRP1 staining in (e). Dots in (f) depict individual samples. (g) The mRNA expression of GATA3 and NRP1 genes was measured by qRT-PCR in (e). Dots in (g) depict individual samples (*n* = 4, ^∗^*P* < 0.05 vs. A549-shNC). (h) Western blotting was used to detect GATA3 and NRP1 proteins in tumors of the four groups.

To directly test whether GATA3 knockdown increases radiosensitivity by regulating NRP1 *in vivo*, nude mice-bearing xenografts from A549-shNC and A549-shGATA3 were treated with or without 20 Gy IR. The xenografted tumors originated from A549 cells with stable low expression of GATA3 genes constructed by subcutaneous injection (Figure S4). Strikingly, GATA3 knockdown combined with IR significantly suppressed A549 tumor growth (Figures 6(a) and 6(b)). However, H1299 cell knockdown GATA3 combined with 20 Gy irradiation did not reduce the tumor (Figure 6(c) and 6(d)). The body weight of nude mice remained essentially unchanged within 14 days after irradiation (Figure S5). IHC results showed that the staining of GATA3 and NRP1 decreased gradually in the four groups (A549-shNC, A549-shNC+20 Gy, A549-shGATA3, and A549-shGATA3+20 Gy) (Figures 6(e) and 6(f)). This indicates that the growth inhibitory effect of tumors is related to the reduction of GATA3 and NRP1. Knockdown of GATA3 combined with high-dose irradiation can inhibit the expression of NRP1. Next, the mRNA (Figure 6(g)) and protein expression levels (Figure 6(h)) of GATA3 and NRP1 in tumor tissues also confirmed our conjecture.

## 4. Discussion

In recent years, more and more studies have shown that GATA3 is closely related to the prognosis of various cancers. GATA3 is one of the most frequently mutated genes in breast cancer, and its mutation affects breast cancer progression [28]. GATA3 can promote breast cancer invasion and metastasis through epithelial-mesenchymal transition (EMT) [29, 30]. In addition, GATA3 is a major transcription factor for T cell differentiation into Th2-type cells [32], which is involved in the immune microenvironment of lung tumors and maintains chemoresistance [31]. However, the mechanism of action of GATA3 in nonsmall cell lung cancer is still unclear, and whether GATA3 is related to radiation resistance has not been reported. Therefore, the present study explored the mechanism of GATA3 in two radiation-resistant cell models of nonsmall cell lung cancer (A549-RR and H1299-RR).

Firstly, according to the different sensitivity of the two lung adenocarcinoma cells to radiation, we used different dose rates to construct the radiation resistance models. After successful construction, it was found that the two cells had different cell cycle arrest. This phenomenon may be caused by different cellular genetic backgrounds. A549 cells are p53 wild-type and H1299 cells are p53 deletion-type [32–34]. It is well known that p53 is a tumor suppressor and can also regulate the metabolic pathways of cancer cells. RBL2/DREAM-mediated Aurora kinase A/B pathway inhibition in p53 WT NSCLC can increase the radiosensitivity of NSCLC [35]. On the other hand, p53 is an important checkpoint in the G1/S phase. The radioresistance of p53null H1299 cells results in cell cycle checkpoint disturbance and a higher tetraploid ratio compared to that of p53wt A549 cells. It has been demonstrated in the literature that two types of lung adenocarcinoma cells (A549 and H1299) have different sensitivities to radiation, and after conventionally fractionated irradiation regimens, the two types of cells exhibit different apoptosis, metabolic activity, and EMT transformation [36]. This fully demonstrates that nonsmall cell lung cancer (NSCLC) treatments should become more personalized according to the status of key protein molecules in tumor tissue [34]. This also coincides with our research. Our study also found that the mechanisms by which the two types of lung cancer cells develop radioresistance are different, and in-depth exploration has been carried out.

After that, we analyzed the difference of gene expression between A549 and A549-RR cells. The results showed that GATA3 and NRP1, a key gene closely related to the high invasion and metastasis of lung cancer [37–39], were highly expressed. A large number of studies have confirmed that the transcription factors GATA3 and NRP1 are involved in the process of EMT. So, what is the relationship between them? Our study found that NRP1 is a direct downstream target gene of GATA3. GATA3 positively promoted NRP1 expression in A549 and A549-RR cells but inhibited NRP1 expression in H1299 and H1299-RR cells (Figure 3). As we all know, the complexity of transcription factor regulation depends in part on its transcription cofactor. GATA3, as a biological regulator of tumor cells, can form complexes with a variety of proteins to jointly regulate the transcription of target genes.

As research progresses, it has become increasingly recognized that both genetic and epigenetic events can contribute to cancer development [40]. Since epigenetic changes are reversible and epigenetic regulators are often proteins with enzymatic activities, which can regulate the expression of many target genes, and depending on different cell environments, they can play different roles as tumor suppressors or oncoproteins [41]. Therefore, we speculate whether there is a histone modification involved in the regulation of NRP1 by GATA3. We analyzed competitive H3K4me3 and GATA3 binding in the NRP1 promoter region by ChIP and ChIP-seq (Figure 5(c)). H3K4me3 modification is a marker of gene activation [42, 43], which can jointly promote the transcription of NRP1 in A549-RR cells. However, the modification of H3K4me3 in H1299-RR cells was less than that in H1299 cells, indicating that the role of H3K4me3 in H1299-RR cells was weakened and GATA3 played a leading role. Finally, we verified the regulatory effect of GATA3 on NRP1 in vivo. GATA3 knockdown in A549 cells increased radiosensitivity of A549 cells and reduced tumor volume (Figure 6(a)).

## 5. Conclusions

**Figure 7:**
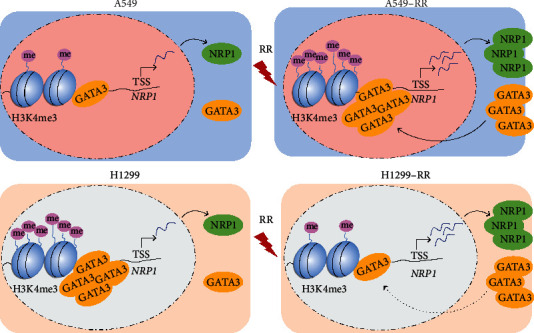
A working model of GATA3/H3K4me3-regulating NRP1-induced radiation resistance in two kinds of nonsmall cell lung cancer cells (A549 and H1299).

In summary, during the formation of radiation resistance in A549 cells, the transcription factor GATA3 will be recruited to the NRP1 gene promoter region. Additionally, the H3K4me3 alteration in the NRP1 gene promoter region will increase, increasing NRP1 gene transcription and resulting in radiation resistance in A549 cells. The process of radiation resistance in H1299 cells, on the other hand, is fundamentally different from that in A549 cells. In H1299 cells, the transcription factor GATA3 acts as a transcription inhibitor to inhibit the transcription of NRP1. Moreover, the enrichment of GATA3 and H3K4me3 in the promoter region of NRP1 gene decreased. Therefore, we speculate that the main factor of radiation resistance in H1299 cells is the increase of NRP1 transcription caused by the decrease of GATA3 enrichment (Figure 7). Overall, our results clarify that GATA3 has different regulatory effects on NRP1 in two lung adenocarcinoma cells, and H3K4me3 is also involved.

## Figures and Tables

**Table 1:** The sequences of the qRT-PCR primers.

qRT-PCR primers		Sequence 5′ to 3′
NRP1	Forward	CCCCAAACCACTGATAACTCG
	Reverse	AGACACCATACCCAACATTCC
GATA3	Forward	AAGGCAGGGAGTGTGTGAAC
	Reverse	CGGTTCTGTCCGTTCATTTT
KMT2B	Forward	TCCATCTTCACTGACCCACC
	Reverse	GTGACGACTGAGGTAGGAGG
GAPDH	Forward	ACGGATTTGGTCGTATTGGG
	Reverse	TGATTTTGGAGGGATCTCGC

**Table 2:** Antibodies used in western blotting.

Antibodies	Catalog number	Company	Dilutions
Anti-GAPDH	TA802519	OriGene	1 : 1000
Antineuropilin 1 antibody	ab81321	Abcam	1 : 1000
Anti-GATA3 antibody	ab199428	Abcam	1 : 1000
Antihistone H3 (tri methyl K4)	ab213224	Abcam	1 : 1000
Antihistone H3	ab1791	Abcam	1 : 1000
Flag-tag (1A8) mAb	AP0007M	Bioworld	1 : 1000
Goat antimouse IgG (H+L) HRP	BS12478	Bioworld	1 : 50000
Goat antirabbit IgG (H+L) HRP	BS13278	Bioworld	1 : 10000
Cy3 goat antibody	AS007	ABclonal	1 : 250
Recombinant rabbit IgG	ab172730	Abcam	1 : 100

**Table 3:** The sequences of the ChIP-qPCR primers.

ChIP-qPCR primers		Sequence 5′ to 3′
NRP1-promoter-primer 1	Forward	CACACTCAGCAGGGAAAGG
	Reverse	GAGCGCCCGTTTGGATAG
NRP1-promoter-primer 2	Forward	GAGCAGTTACCATCCAGTCTAC
	Reverse	TAGGAGGTGCTGCAGAAATAAG

## Data Availability

The data used to support the findings of this study are available from the corresponding authors upon request.

## Supplementary Materials

Supplementary Materials Table S1: the sequences of the siRNA oligonucleotides and the shRNA. Table S2: the *D*_0_, *n*, *D*_*q*_, SF_2_, and *R*^2^ values of the single-click multitarget model. Figure S1: the expression of GATA3 in A549 and A549-RR cells transfected with siRNAs. Figure S2: GATA3 positively regulates NRP1 expression in A549 and A549-RR cells. Figure S3: GATA3 represses NRP1 expression in H1299 and H1299-RR cells. Figure S4: establishment of A549 cell model knockdown GATA3. Figure S5: the average body weights of the mice among groups.
