# Supplementary material for: GATA3 Exerts Distinct Transcriptional Functions to Regulate Radiation Resistance in A549 and H1299 Cells
Source: Oxid Med Cell Longev. 2022 Aug 10;2022:9174111. doi: 10.1155/2022/9174111 (PMC9385326; doi:10.1155/2022/9174111)

**Supplementary Materials**

**Table S1: The sequences of the si-RNAs oligonucleotides and the sh-RNAs.**

| si-NC | sense | UUCUCCGAACGUGUCACGUTT |
| --- | --- | --- |
|  | antisense | ACGUGACACGUUCGGAGAATT |
| si-GATA3-1 | sense | UCUGGAGGAGGAAUGCCAATT |
|  | antisense | UUGGCAUUCCUCCUCCAGATT |
| si-GATA3-2 | sense | CCCUGACUAUGAAGAAGGATT |
|  | antisense | UCCUUCUUCAUAGUCAGGGTT |
| si-GATA3-3 | sense | ACGAGAAAGAGUGCCUCAATT |
|  | antisense | UUGAGGCACUCUUUCUCGUTT |
| sh-NRP1 | sense | GCCAGAGGAGTACGATCAGTT |
| Sh-GATA3 | sense | CGCTGGGTGAGCCACCACCA |

**Table S2: The D_0_, n, D_q_, SF_2_ and R^2^ value of single-click multi-target model.** Differences between experimental groups were assessed by Student’s t-test or one-way analysis of variance. Data represent mean ± SD. ^*^*p*<0.05 vs A549, ^#^*p*<0.05 vs H1299 (n=3).

| Group | D_0_ | n | D_q_ | SF_2_ | R^2^ |
| --- | --- | --- | --- | --- | --- |
| A549 | 1.85 | 1.79 | 1.08 | 0.42±0.12 | 0.99 |
| A549-RR | 1.66 | 5.67 | 2.9 | 0.83±0.02^*^ | 1.00 |
| H1299 | 1.26 | 2.76 | 1.27 | 0.47±0.09 | 1.00 |
| H1299-RR | 3.68 | 1.50 | 1.49 | 0.74±0.02^#^ | 1.00 |

**Figure S1: The expression of GATA3 in A549 and A549-RR cells transfected with siRNAs.** Three different siRNAs targeting GATA3 (1-3) were transduced into A549 and A549-RR cells. The mRNA expression of GATA3 was measured by qRT-PCR, and GAPDH was used as internal control. Data were represented as means ± SD from three independent experiments. (n=3, ^*^*P*<0.05 vs A549, ^#^*P*<0.05 vs A549-RR, two-tailed t-test)

**
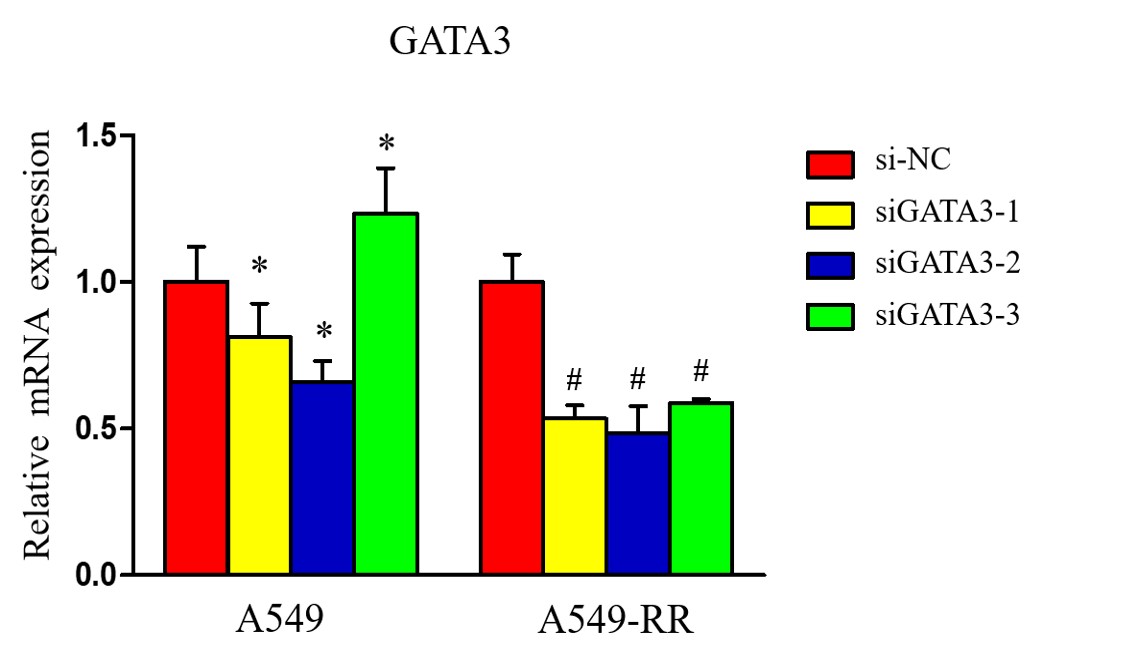
**

**Figure S2:** **GATA3 positively regulates NRP1 expression in A549 and A549-RR cells.** **(a)** The mRNA expression of GATA3 and NRP1 involved in A549 and A549-RR cells transfected with negative control si-NC or si-GATA3. **(a)** The mRNA expression of GATA3 and NRP1 involved in A549 and A549-RR cells transfected with vector or pcDNA3.1(+)-GATA3. **(c)** The mRNA expression of GATA3 and NRP1 involved in A549 and A549-RR cells transfected with negative control sh-NC or sh-NRP1. **(d)** The mRNA expression of GATA3 and NRP1 involved in A549 and A549-RR cells transfected with vector or pLNCX2-NRP1. Results are represented as fold change over control, with GAPDH used as the internal reference. Data were shown as means ± SD from three independent experiments (n=3, ^*^*P*<0.05 vs A549, ^#^*P*<0.05 vs A549-RR, two-tailed t-test).


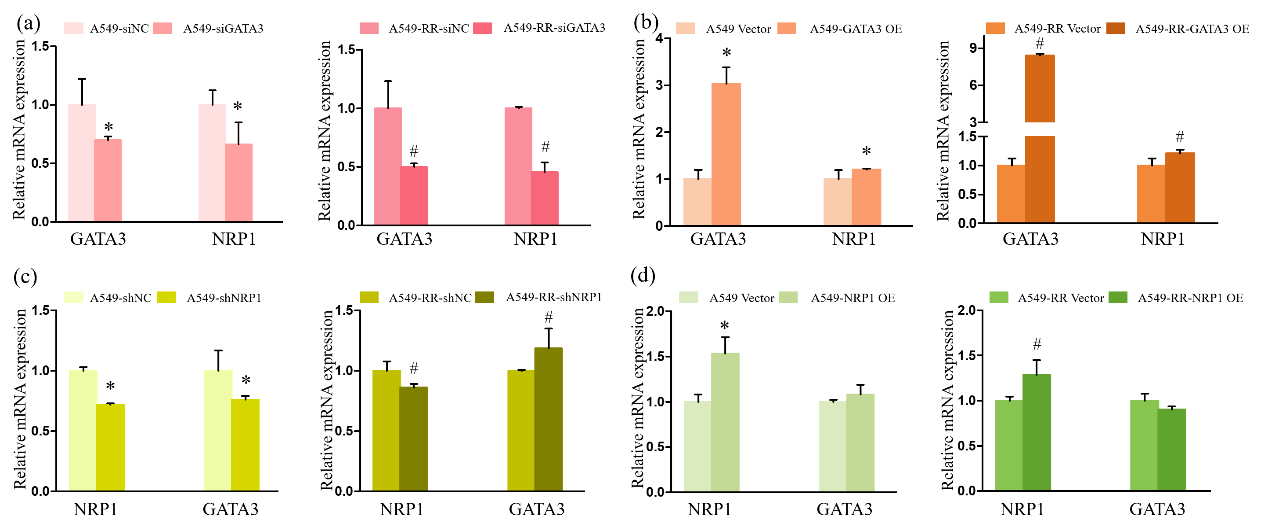


**Figure S3:** **GATA3 represses NRP1 expression in H1299 and H1299-RR cells.**

**(a)** The mRNA expression of GATA3 and NRP1 involved in H1299 and H1299-RR cells transfected with negative control si-NC or si-GATA3. **(b)** The mRNA expression of GATA3 and NRP1 involved in H1299 and H1299-RR cells transfected with vector or pcDNA3.1(+)-GATA3. **(c)** The mRNA expression of GATA3 and NRP1 involved in H1299 and H1299-RR cells transfected with negative control sh-NC or sh-NRP1. **(d)** The mRNA expression of GATA3 and NRP1 involved in H1299 and H1299-RR cells transfected with vector or pLNCX2-NRP1. Results were represented as fold change over control, with GAPDH used as the internal reference. Data were shown as means ± SD from three independent experiments (n=3, ^*^*P*<0.05 vs H1299, ^#^*P*<0.05 vs H1299-RR, two-tailed t-test).


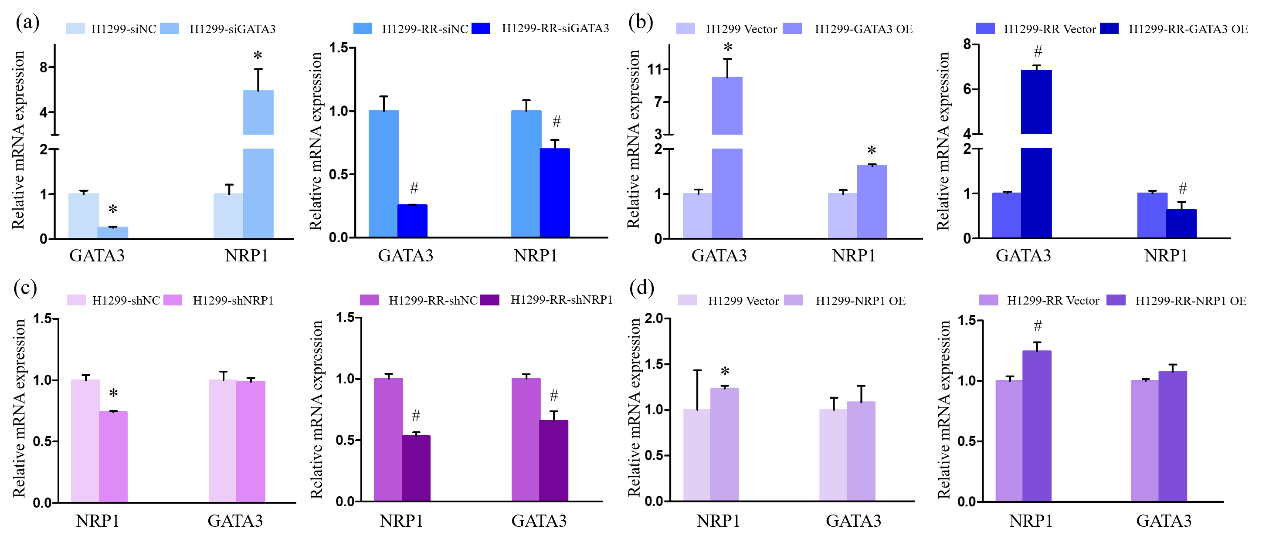


**Figure S4: Establishment of A549 cell model knockdown GATA3.**

**(a)** Intracellular fluorescence intensity was measured after 7 days of lentiviral transduction of shRNA targeting *GATA3*. **(b)** The shRNA targeting *GATA3* were transduced into A549 cells. The mRNA expression of GATA3 and NRP1 were analyzed by qRT-PCR. GAPDH was used as internal control. Data were represented as means ± SD from three independent experiments. (n=3, ^*^*P*<0.05 vs A549, two-tailed t-test) **(c)** Protein level of GATA3 and NRP1 were analyzed by Western blot. GAPDH was used as internal control.


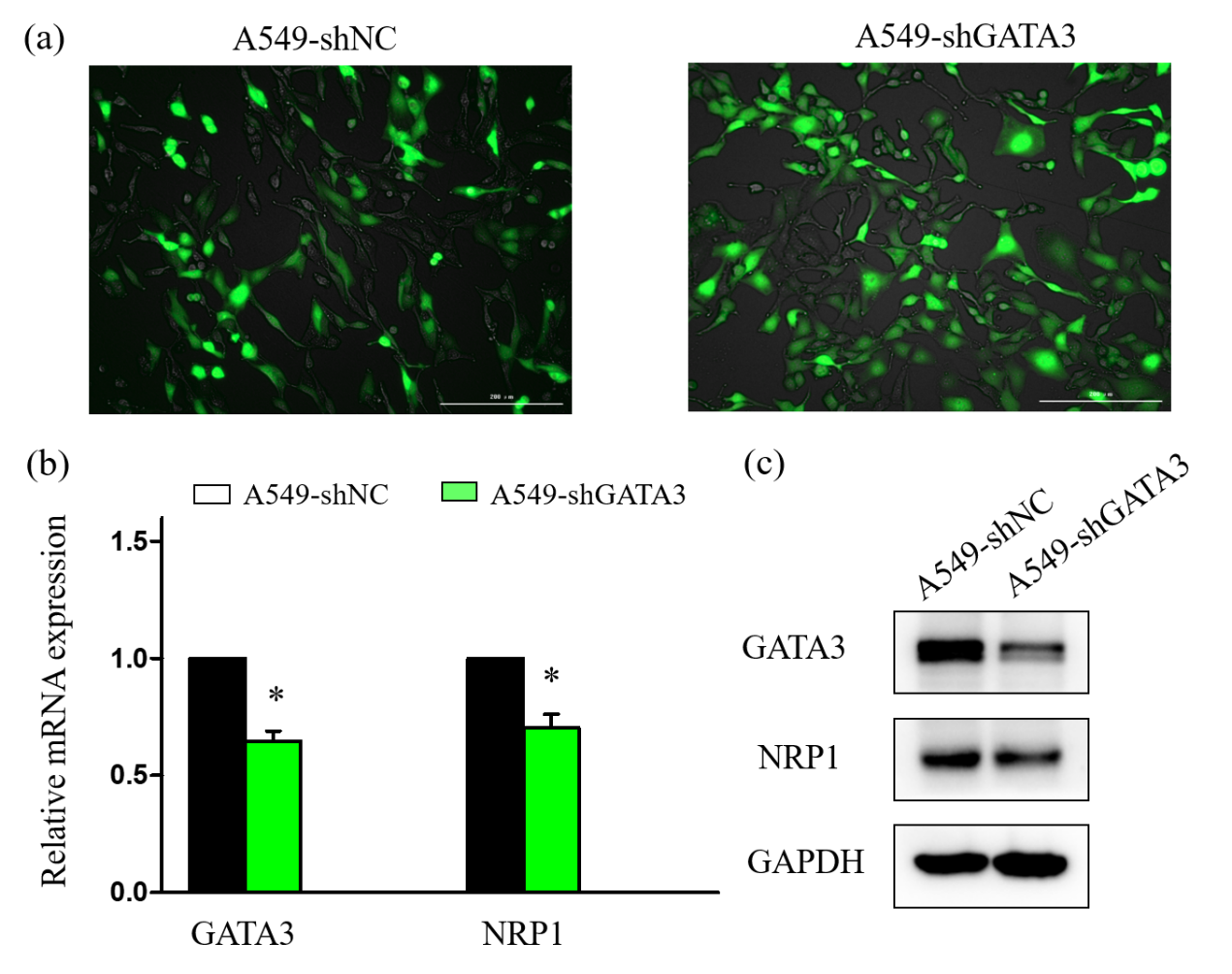


**Figure S5: The average body weights of the mice among groups.**

**(a) and (b)** The average body weights of mice among groups were examined every 2 days. Data were shown as means ± SD.


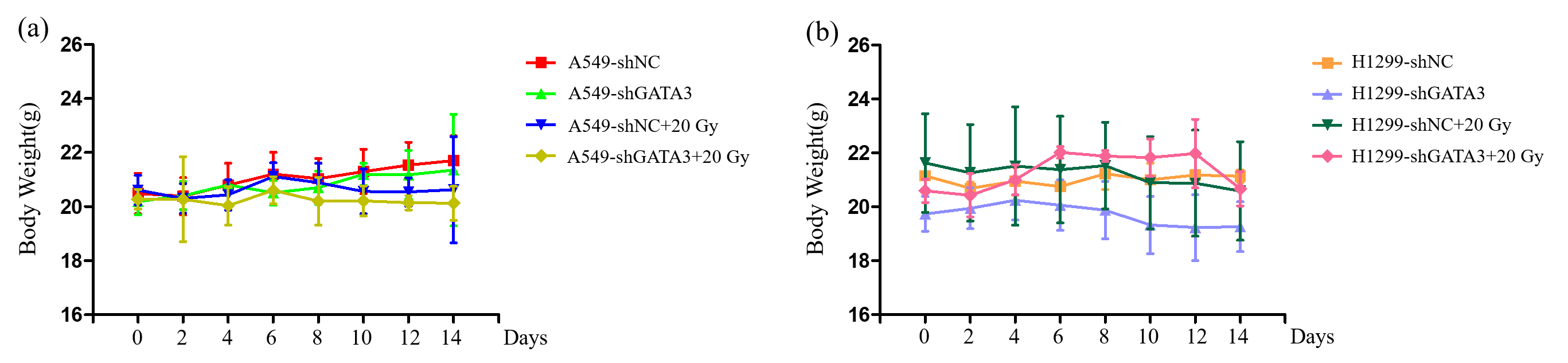

Supplement: Supplementary Materials — Table S1: the sequences of the siRNA oligonucleotides and the shRNA. Table S2: the D0, n, Dq, SF2, and R2 values of the single-click multitarget model. Figure S1: the expression of GATA3 in A549 and A549-RR cells transfected with siRNAs. Figure S2: GATA3 positively regulates NRP1 expression in A549 and A549-RR cells. Figure S3: GATA3 represses NRP1 expression in H1299 and H1299-RR cells. Figure S4: establishment of A549 cell model knockdown GATA3. Figure S5: the average body weights of the mice among groups. [file 9174111.f1.docx]
